# New Insights into the Virulence Traits and Antibiotic Resistance of Enterococci Isolated from Diverse Probiotic Products

**DOI:** 10.3390/microorganisms9040726

**Published:** 2021-03-31

**Authors:** Fengru Deng, Yunsheng Chen, Xiaoyu Zhou, Huiying Xiao, Tianyu Sun, Yiqun Deng, Jikai Wen

**Affiliations:** 1 Guangdong Provincial Key Laboratory of Protein Function and Regulation in Agricultural Organisms, College of Life Sciences, South China Agricultural University, Guangzhou 510642, Guangdong, China; dengfengru@scau.edu.cn (F.D.); yunshengchen@stu.scau.edu.cn (Y.C.); 20202003036@stu.scau.edu.cn (X.Z.); huiyingxiao@stu.scau.edu.cn (H.X.); suntianyu@stu.scau.edu.cn (T.S.); 2 Key Laboratory of Zoonosis of Ministry of Agriculture and Rural Affairs, South China Agricultural University, Guangzhou 510642, Guangdong, China; 3 Guangdong Laboratory for Lingnan Modern Agriculture, South China Agricultural University, Guangzhou 510642, Guangdong, China

**Keywords:** probiotics, enterococci, hemolysis, virulence factors, biofilm, antimicrobial resistance, transposon ICE

## Abstract

The GRAS (generally recognized as safe) status of *Enterococcus* has not yet been authenticated, but enterococci, as probiotics, have been increasingly applied in human healthcare and animal husbandry, for instance as a dietary supplement, feed additive, or growth promotor. The food chain is the important route for introducing enterococci into the human gut. The pathogenicity of *Enterococcus* from probiotic products requires investigation. In the study, 110 commercial probiotic products used for human, animal, aquaculture, and plants were examined, among which 36 enterococci were identified, including 31 from *Enterococcus faecium*, 2 from *E. faecalis*, 2 from *E. casseliflavus*, and 1 from *E. gallinarum*. Strikingly, 28 of the 36 enterococci isolated from probiotics here did not mention the presence of *Enterococcus* in the labeled ingredients, and no *Enterococcus* isolates were found from 5 animal probiotics that were labeled with the genus. In total, 35 of the 110 products exhibited hemolysis, including 5 (10.6%) human probiotics, 14 (41.2%) animal probiotics, 8 (57.1%) aquaculture probiotics, and 8 (53.3%) plant probiotics. The detection rates of virulence factors associated with adhesion, antiphagocytosis, exoenzyme, biofilm, and other putative virulence markers (PVM) in 36 enterococci were 94.4%, 91.7%, 5.6%, 94.4% and 8.3%. Twenty-six of the 36 isolated strains exhibited biofilm formation ability, where 25 strains (69.4%) and one (2.8%) were strong and weak biofilm producers, respectively. We analyzed the resistance rates against erythromycin (97%), vancomycin and ciprofloxacin (8%), tetracycline (3%), and high-level aminoglycosides (0%), respectively. High detection rates of *msrC/lsaA* (86%) and *aac(6′)-Ii* (86%) were observed, followed by *vanC* (8%), *tetM* (3%). The Tn*5801*-*tetM*-like integrative conjugative element (ICE) was identified in *E. gallinarum*, exhibiting resistance to tetracycline (64 μg/mL). Seven probiotic *E. faecalis* and *E. faecium*, as active ingredients in human probiotics, shared the same STs (sequence types) and were distinct from the STs of other contaminated or mislabeled enterococci, indicating that two particular STs belonged to native probiotic isolates. These findings advocate appropriate assessments of enterococci when used in probiotics.

## 1. Introduction

In the last few years, probiotics have been increasingly used in human, animal, aquaculture, and plant health worldwide. Probiotics are not only applied for healthcare, feed additive growth promotion and so on, but are also emerging as novel therapeutic tools for treating diseases such as FGIDs (functional gastrointestinal diseases) and septicemia [1,2]. The probiotic market in America is the most advanced, with China and Japan having occupied over half of the Asia-Pacific probiotic sales for decades.

A probiotic is a live microorganism isolated from different genera and species and is generally recognized as safe (GRAS) and effective. Among numerous microorganisms, lactic acid bacteria (LAB) and *Bifidobacterium* are the most popular and widely utilized as probiotic candidates worldwide. Enterococci, one kind of LAB, are commensal organisms that are well suited to survival in the gastrointestinal tract of human and animal and environments like water and soil. Enterococci have been identified as opportunistic pathogens that cause various infections, among which approximately 80% are associated with *Enterococcus faecalis* [3]. More importantly, the emergence and spread of vancomycin-resistant *Enterococcus* (VRE) isolates presents serious therapeutic difficulty, owing to a lack of effective antimicrobial therapy [4]. In fact, quite a few enterococcal strains have been authorized as probiotics for use in pharmaceutical preparations or animal feed additives for decades, such as *E. faecium* Medilac-Vita (treatment of infantile enteritis, China), *E. faecium* SF68^®^ (dietary supplementation for human and animal, Switzerland), *E. faecium* Cylactin^®^ (feed additive for animal, Switzerland), *E. faecalis* Symbioflor^®^ 1 (treatment of respiratory illness, Germany), and *E. faecalis* TH10 Dr. Ohhira^®^ (dietary supplementation for human, Republic of Estonia). However, the FDA (Food and Drug Administration) of Taiwan, Province of China, has restricted the use of *E. faecium* and *E. faecalis* as food supplements in probiotics since July of 2018, owing to the widespread of multi-resistant enterococci, irrespective of origins and the isolated location. As *Enterococcus* has not yet been authenticated with the status of GRAS by the FDA, a limited number of enterococci as probiotics have been commercialized [5]. The mainly controversial issue of enterococci use in probiotics is the risk of their pathogenicity, i.e., virulence genes and antibiotic resistance genes (ARGs) of this genus being transferred horizontally to commensal gut microflora via the food chain, and the lack of legislation [4,5].

In this study, we analyzed the contamination, label accuracy, and hemolysis of commercial probiotic products for human, animal, aquaculture, and plant use. The hemolytic activity, cytotoxicity, virulence factors, biofilm formation, antimicrobial resistance, and molecular types of enterococci originating from these various products are analyzed. These findings provide new scientific insights into the safety assessment of probiotic products and enterococcal isolates.

## 2. Materials and Methods

### 2.1. Isolation and Identification of Enterococcus spp. from Probiotic Products

The commercial probiotic products were collected between 2018–2020 (Supplementary Table S1). After the solid probiotic products were fully ground, 2 g of probiotic powder and 2 mL of the liquid probiotic product were suspended in 20 mL of sterile phosphate buffered saline (PBS). The probiotic suspensions were cultured in the 500 μL brain heart infusion (BHI) broth containing 6.5% NaCl and were incubated at 37 °C for 8 h for pre-enrichment, and the inoculum size in the broth was 2%. Afterwards, the bacterial cultures were spread on *Enterococcus*-selective media (Beijing Land Bridge Technology, Beijing, China) and incubated at 37 °C for 18 h. Brown single colonies of each distinct phenotype were sub-cultured in 1 mL BHI broth. Thereafter, the overnight cultures were diluted and spread-plated onto agar plates so that the isolated colonies could be obtained for further study.

Genomic DNAs were extracted from isolates using the TIANamp Genomic DNA Kit (Tiangen Biotech, Beijing, China), and the 16S rDNA gene was amplified using bacterial universal primers 27F and 1492R. The obtained sequences were analyzed using the nucleotide basic local alignment search tool (BLAST) [6] at NCBI for further identification of isolates from the probiotic products.

### 2.2. Toxin Production, Hemolysis Detection, and Cytotoxicity Assays

Hemolysis tests and cytotoxicity assays were performed in order to assess the biological safety of both the probiotic products and their *Enterococcus* isolates. After suspending 2 g of probiotic powder and 2 mL of the liquid probiotic in 20 mL of PBS, suspensions were cultured in 10mL lysogeny broth (LB) medium at 30 °C, and the inoculum size in the broth was 2%. Meanwhile, *Enterococcus* isolates were cultured with an identical inoculum size in the broth. Both overnight supernatant cultures of probiotic products and isolates were harvested by centrifugation at 13,000 rpm for 5 min and stored at −20 °C until used for toxin production analysis.

The hemolysis of each probiotic product and isolate was determined according to the method described previously with minor modification [7]. Briefly, sheep red blood cells (SBCs) (Hongquan Bio, Guangzhou, China) were harvested by centrifugation at 3000 rpm for 10 min, followed by rinsing twice with PBS. After 8 vol % suspensions of SBCs were prepared using PBS, the suspensions were mixed with equal volumes of culture supernatants to generate final suspensions of 4% (vol/vol) SBCs, and then were incubated at 37 °C for 1 h, then finally harvested by centrifugation at 3000 rpm for 10 min at 4 °C. The 100-μL concentrate sample in each tube was transferred to a sterile 96-well plate and the hemolytic activity was assessed by measuring the optical absorbance at OD_576_ with a microplate absorbance spectrophotometer (Bio-Rad, Hercules, CA, USA). Human red blood cells (RBCs) (Hongquan Bio, Guangzhou, China) were harvested by centrifugation for 3000 rpm for 10 min, followed by rinsing twice with PBS. After the suspensions of RBCs were prepared using PBS, the suspensions and molten LB agars (50 °C) were mixed in sterile culture dishes to generate final agars of 5% (vol/vol) RBCs. The overnight cultures of isolates were sub-cultured via streaking on RBC agar plates and were incubated at 37 °C for 48 h. Each plate was checked for single colony growth and assessment of the hemolytic zone.

The 10 μL culture supernatants (for toxin production) of the isolates were used to test the cytotoxicity on Vero cells as previously described [8]. The water-soluble tetrazolium salt-8 (WST-8, MCE) method and a microscope were also used to determine whether the isolates were cytotoxic. Vero cells were obtained from Professor Kui Zhu at China Agricultural University. All tests were repeated three times and the significant difference was calculated.

### 2.3. Antimicrobial Susceptibility Test

The microdilution broth method, as described by the CLSI (Clinical Laboratory and Standards Institute) documents [9,10], was used to determine the susceptibility of *Enterococcus* to 11 antimicrobial agents, including ampicillin, erythromycin, tetracycline, tigecycline, ciprofloxacin, gentamicin, streptomycin, linezolid, florfenicol, vancomycin, and teicoplanin. The MIC (minimum inhibitory concentration) ranges of the antimicrobial agents and the resistance breakpoints of the antimicrobial agents were found via referral to the CLSI documents.

### 2.4. Whole-Genome Sequencing

The whole genome of *Enterococcus* was sequenced on an Illumina NovaSeq 6000 platform with the 150 bp paired-end module, and the sequencing library was generated using the VAHTS Universal DNA Library Prep Kit for Illumina^®^ (Vazyme ND604, Nanjing, China) following manufacturer’s recommendations. High-quality reads were de novo assembled using the SPAdes software v3.1.0 and annotated using the Prokka v1.12.0.

In order to screen isolates for the presence of virulence factors and ARGs, the core genome tree of *Enterococcus* was constructed using panX (pan-genome-analysis pipeline), and the whole genome of *Enterococcus* was analyzed using Blastn v2.11.0 (identity ≥80% and coverage ≥80%) via the following databases, respectively: Virulence Factors of Pathogenic Bacteria (VFDB) (accessed on 10, Dec 2020) [11], the reported virulence factors [12], ResFinder v4.0 [13], and PointFinder v4.0 [14]. MLST (multilocus sequence typing) of the seven housekeeping genes (*gdh*, *gyd*, *pstS*, *gki*, *aroE*, *xpt*, and *yiqL*) for *Enterococcus* was performed with *Enterococcus* MLST databases [15]. The allelic profiles and the sequence types were generated by using the BLAST with the *Enterococcus* sequences in the MLST database (https://pubmlst.org/databases/ (accessed on 10 December 2020)).

### 2.5. Biofilm Assay

Crystal violet assay biofilm mass was quantified using the crystal violet assay as previously described [16]. *E. faecalis* JH2-2 was used as the quality control strain.

### 2.6. Statistical Analysis

GraphPad Prism version 8.3 was used for all statistical analysis. Hemolysis products were tested with the unpaired t-test. Statistical significance was determined and recorded as follows: *p* < 0.001 (***), *p* < 0.01 (**), *p* < 0.05 (*), or not statistically significant if *p* > 0.05.

## 3. Results and Discussion

### 3.1. Identification of Enterococci, Contamination, and Label Inaccuracy

**Figure 1:**
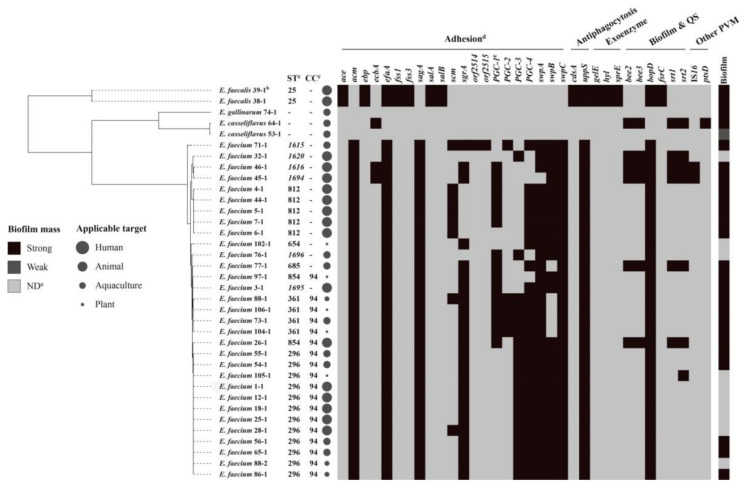
Virulence genes, MLST (multilocus sequence typing), and biofilm mass profiles of 36 *Enterococcus* spp. isolates from the probiotics. Note: (a) ND, no biofilm mass; PVM, putative virulence marker. (b) The rule of strain name is the first number and represents the originated probiotic product, and the latter number represents the isolated strain. (c) CC, clonal complex; the new STs (sequence types) were labelled in italic; -, ST or clonal complex cannot be assigned. (d) In the heatmap, black color indicates strain carrying the corresponding virulence gene. (e) PGC-1, a complete PGC-1 cluster, including *pilA*, *pilE*, *pilF*, *orf1903*, *orf1905*, and *orf1916*; PGC-2, a complete PGC-2 cluster, including *orf2008*, *orf2009*, and *orf2010*; PGC-3, a complete PGC-3 cluster, including *pilB*, *pilE*, *pilF*, *orf2568*, *orf2570*, and *orf2571*.

The products were collected from South Korea, Australia, America, The Netherlands, and 21 provinces/municipalities/autonomous regions (P/M/A) in China (Supplementary Table S1), covering more than half of the provincial administrative regions in China. In total, 110 probiotic products, including 47 human products used for preventive care and treatment, 34 animal products used for precaution, therapy, food additive, and excreta degradation, 14 aquaculture products used for water purification, and 15 plant products used for biocontrol and growth promotion, were examined. The active ingredients of all products are described in Supplementary Table S1. Based on the colony morphologies of selected media and 16S rDNA gene sequences from the whole genome sequence analysis, 36 *Enterococcus* spp. (31 *E. faecium*, 2 *E. faecalis*, 2 *E. casseliflavus*, and 1 *E. gallinarum*) were isolated and identified from 110 probiotic products (Figure 1 and Supplementary Table S1).

Among all 13 products labeled with ingredients containing *Enterococcus* spp., no *Enterococcus* was isolated from five animal products (No. 61, 63, 72, 78, and 79), while one *E. faecium* isolate and one *E. gallinarum* isolate were identified in two animal products (No. 65 and 74) labeled with ingredients containing *E. faecalis*. Over half of the products labeled with ingredients containing *Enterococcus* spp. were below the standard. This result is in accordance with previous findings [17,18] where the active ingredient was missing in quite a few probiotic products.

Furthermore, 26 *E. faecium* and 2 *E. casseliflavus* isolates were identified in 27 products (from all four origins) labeled with ingredients not containing *Enterococcus*, suggesting that these products were contaminated by these two species. Compared with *E. faecalis*, *E. faecium* has a high frequency of multi-resistant phenotypes and composes the majority of VRE infections [19]. Interestingly, *E. casseliflavus* and *E. gallinarum*, which are not authorized for use in probiotics, were present in two probiotic products. Besides, the overwhelmingly predominant species among enterococci, originating from diverse sources, are *E. faecalis* and *E. faecium* [20,21]. Therefore, it is unknown how these probiotic products were contaminated by *E. casseliflavus* and *E. gallinarum*. Since enterococci are widespread in the intestinal tracts of mammals and birds, and also in water and soil [20], this could explain the possibility of cross contamination by different *Enterococcus* sp. that are not listed as an ingredient in the probiotics considered here. Usually, the isolation rates of *E. faecalis* are higher than those of *E. faecium*, irrespective of the sample origin [4,20], while *E. faecium*, with an isolation rate of 86%, was the predominant species in this study. Inappropriately, quite a few probiotic products are directly labeled with ingredients containing LAB, thereby causing confusion, since the term “LAB” includes several genera, such as *Lactobacillus*, *Streptococcus*, *Pediococcus*, and *Enterococcus*. Label inaccuracy for products from all four origins was also observed.

Together, these findings are in accordance with previous studies [17,18,22] and confirm that the cross contamination, label inaccuracy, and lack of active ingredients in probiotic products, especially those for human use, could pose great risks to the health of humans and livestock.

### 3.2. Hemolytic Activity and Cytotoxicity

**Figure 2:**
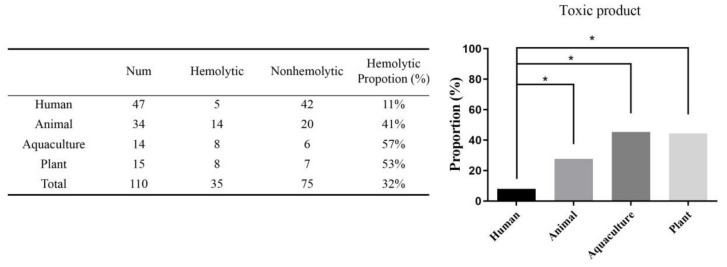
The proportion of toxic products in 110 probiotic products from four origins. Among 110 probiotic products, 35 (31.8%) showed hemolysis, including 5 from human products (4.6%), 14 from animal products (12.7%), 8 from aquaculture products (7.3%), and 8 from plant products (7.3%). Simultaneously, 5 of 47 human products (10.6%) exhibited hemolysis. In total, 14 of 34 animal products (41.2%) exhibited hemolysis, while over one half of aquaculture and animal products exhibited hemolysis. The human products showed significant difference (* *p* < 0.05) in the hemolysis compared with the products of the other three origins.

As depicted in Figure 2, among 110 probiotic products, 35 (31.8%) showed hemolysis, including 5 from human products (4.6%), 14 from animal products (12.7%), 8 from aquaculture products (7.3%), and 8 from plant products (7.3%). In detail, 5 of 47 human products (10.6%) exhibited hemolysis, with a maximum hemolysis rate of 93% for product No. 28. Fourteen of 34 animal products (41.2%) exhibited hemolysis, with a maximum hemolysis rate of 95% for product No. 48, while over one half of the aquaculture and animal products exhibited hemolysis. The human products seem to be relatively secure, showing significant differences (*p* < 0.05) in hemolysis when compared with products of the other three origins.

Although most of hemolytic isolates belong to *E. faecalis* and *E. faecium*, the leading species in enterococcal infections, our results showed that no hemolysis or cytotoxicity was observed for 36 *Enterococcus* spp., indicating that these *Enterococcus* isolates were not responsible for the hemolysis activity of the products. There are different kinds of bacteria and compounds that can cause hemolysis, such as *Bacillus*, which is used in probiotics for humans and animals, showing cytotoxicity owing to *Bacillus* carrying the heat-labile enterotoxins Nhe and Hbl [22,23]. To rule out the possibility of enterococci belonging to the LAB group (also those that like to produce acid) producing hemolytic toxins, both the probiotic products and enterococci were cultured in LB media instead of lactobacilli MRS broths, and the pH values of each culture were confirmed overnight. All cultures with a final pH between 6.0–8.0 were used for hemolysis detection. As a result, the hemolysis activity was caused by the products themselves, such as exotoxins secreted by culturable bacteria or/and endotoxins released when bacteria were ruptured or disintegrated. Nevertheless, the other ingredients in the product, or even contaminants existing in the product, could contribute to the hemolysis as cryptic virulence factors. As shown in Supplementary Table S1, over half of the products considered here contained more than one bacterial species and the frequency of hemolysis was 60.0% in products with genera of more than one class, and this was higher than those with a single genus. Moreover, microflora in probiotic bacteria cultures are likely to play a synergistic role in toxic substances. Based on these observations, it seems difficult to assess what potential hemolytic factors might exist for probiotic products. Whole-process supervision is the most important and effective measure to ensure the safety of probiotics.

### 3.3. Virulence Factors Associated with Adhesion, Antiphagocytosis, Exoenzymes, and Biofilm Mass

**Figure 3:**
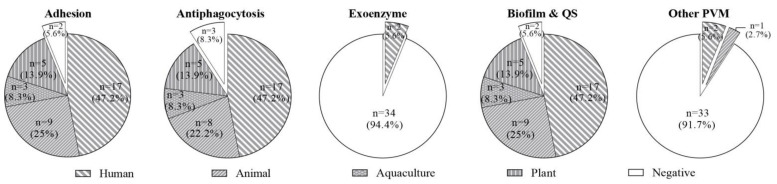
The virulence profile of 36 *Enterococcus* spp. isolates from four origins. The detection rates of virulence factors associated with adhesion, antiphagocytosis, exoenzyme, biofilm, and other PVM in 36 *Enterococcus* spp. were 94.4%, 91.7%, 5.6%, 94.4%, and 8.3%. In the above diagrams, negative indicates an isolate not carrying the corresponding virulence gene. Except for factors associated with exoenzymes merely detected in two probiotic *E. faecalis* from human products (5.6%), prevalence rates of factors associated with adhesion, antiphagocytosis, and biofilm in enterococci from human, aquaculture, and plant probiotics were 47.2%, 8.3%, and 13.9%, respectively, while these three factors in enterococci from animal probiotics were 25%, 22.2%, and 25%.

Although no toxic phenotypes of the isolates were observed in terms of hemolysis and cytotoxicity, the virulence genotypes of 36 *Enterococcus* spp. were determined. A brief introduction of virulence factor functions is described in Supplementary Table S2. Except for the factors associated with exoenzymes only detected in two probiotic *E. faecalis* from human products (5.6%), the virulence factors associated with adhesion (94.4%), antiphagocytosis (91.7%), biofilm formation (94.4%), and other PVM (8.3%) were extensively identified in enterococci isolated from human, animal, aquaculture, and plant probiotics (Figure 3). The prevalence rates of factors associated with adhesion, antiphagocytosis, and biofilm formation in enterococci from human, aquaculture, and plant probiotics were 47.2%, 8.3%, and 13.9%, respectively, while these three factors in enterococci from animal probiotics were 25%, 22.2%, and 25%, respectively. Interestingly, all enterococci isolates did not cause hemolysis or cytotoxicity. It could be explained because no exotoxin factor, such as cytolysin, resulting in lysing erythrocytes and gram-positive bacteria, was observed in 36 *Enterococcus* spp.

After screening each isolate for the presence of major virulence genes, the results showed that *efaA*, *BopD*, and *uppS* were all detected in *E. faecium* and *E. faecalis* (Figure 1). This was evidenced by the widespread occurrence of these virulence factor-associated proteins in enterococci, whereas 28 virulence factors were rarely detected in *E. casseliflavus* and *E. gallinarum*. Similarly, numerous virulence factors in the genus were mainly found in species of *E. faecalis* and *E. faecium* isolates from diverse sources, except from probiotic products [24]. It seems that *ace*, *ebp*, *fss*, *salA*, *salB*, *cdsA*, *fsrC*, and three exoenzyme factors were restricted to *E. faecalis* here. In all *E. faecalis*, genes of gelatinase (GelE), serine protease (SprE), and hyaluronidase were active, so these may contribute to host tissue invasion. Because hyaluronidase produced by enterococci isolated from patients with abscesses has been described as a spreading factor for worms [25], this supports the importance of this exoenzyme in the infection process. Especially, the probiotic isolates *E. faecalis* 38-1 and 39-1 were indicated as the active ingredients in human probiotic products No. 38 and 39 (Supplementary Table S1), rather than contaminants, and this represents a significant threat to the health of food and humans because of this positive genotype.

Genes associated with biofilm formation and quorum sensing (QS) were found in 34 *Enterococcus* spp. (94.4%). The Gene *bopD*, involved in biofilm production, was widespread in *E. faecium*. Gene encoding sortase (Srt), which is important for the biofilm production of *E. faecalis*, has been isolated from a patient with endocarditis [26] and has been found in 19% of enterococci. Simultaneously, gene encoding endocarditis and biofilm-associated pili (Ebp pili, *ebp*), which are not only important for adherence to host extracellular matrix proteins (including fibrinogen and collagen), but also play an important role in endovascular infection [26], were also found in probiotic *E. faecalis* of human probiotic products No. 38 and 39. By contrast, the *bee* gene (biofilm enhancer in *Enterococcus*), which has been confirmed to confer a high biofilm-forming phenotype to *E. faecalis* [27], was found in five isolates of *E. faecium* and one isolate of *E. casseliflavus* here.

The biofilm formation of enterococci represents a critical safety issue in both the healthcare field and the food industry, since *E. faecalis* isolated from endocarditis produces biofilms significantly more often than nonendocarditis *Enterococcus* [28]. In total, 26 of 36 enterococci (72.2%) exhibited an ability of biofilm formation, where 69.4%, 0%, and 2.8% of them were strong, moderate, and weak biofilm producers (Table 1). The enterococci isolated from animal probiotics showed a significantly higher frequency of strong biofilm formation when compared with enterococci isolated from the other three origins. Notably, among 36 enterococci, two *E. faecalis* (38-1 and 39-1) and four *E. faecium* (4-1, 5-1, 6-1, and 7-1) strongly formed biofilms and were all listed as active ingredients in human probiotics. The widespread presence of the biofilm phenotype in enterococci, irrespective of origin, suggests interspecies transmission of biofilm-associated genes.

The food chain has been considered as an important source for the dissemination of enterococci to humans and animals. In many cases, the oral administration of probiotic *Enterococcus* carrying potential virulence factors for patients with immunodeficiency syndrome, diarrhea, or IBD (inflammatory bowel disease) may cause significant infections and diseases, such as endocarditis and bacteremia [4]. Based on these observations, we speculate that the production of different virulence factors may be beneficial for the survival of *Enterococcus* spp. in diverse environments and is likely to contribute to enterococci being the predominant contaminants during production processes for probiotic products. For a Qualified Presumption of Safety (QPS) status, the EFSA (European Food Safety Authority) requires demonstration of the absence of virulence factors of probiotic strains. Adhesion, hemolysin, hyaluronidase, biofilms and so on are putative virulence factors which should be included in *Enterococcus* because they are likely to be transferred to the gut microflora of humans and animals via the oral administration of probiotics, and eventually contributing to the environmental reservoir of virulence factors.

### 3.4. Antimicrobial Resistance and Genetic Environment of tetM

**Figure 4:**
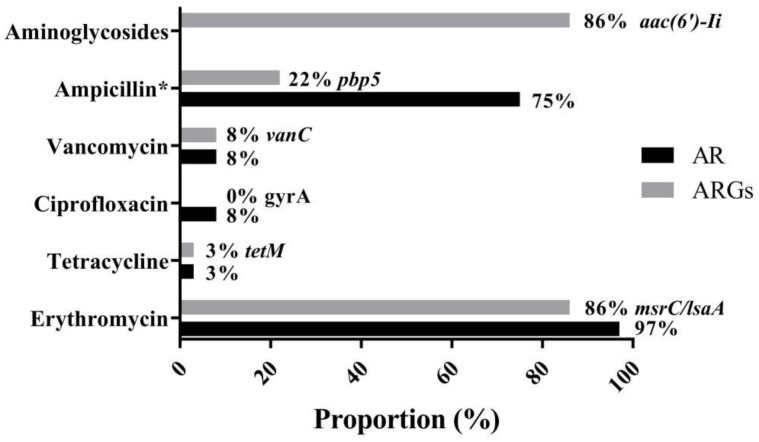
Major antimicrobial resistance phenotype (AR, antibiotic resistance) and genotype (ARGs, antibiotic resistance genes) in 36 enterococci. Resistance rates against erythromycin (97%), vancomycin and ciprofloxacin (8%), tetracycline (3%), and aminoglycosides (0%) were found in 36 enterococci, respectively. * indicates an isolate with an MIC of ampicillin of >2 μg/mL. High detection rates of *msrC*/*lsaA* (86%), *aac(6′)-Ii* (86%), followed by *pbp5* (22%), v*anC* (8%), *tetM* (3%), and ciprofloxacin resistance gene (0%) were observed, respectively.

The MIC breakpoints for ampicillin, erythromycin, tetracycline, tigecycline, ciprofloxacin, linezolid, florfenicol, vancomycin, and teicoplanin are 16, 8, 16, 0.25, 4, 8, 32, 32, and 32 μg/mL, respectively. As shown in Figure 4 and Supplementary Table S3, 35 of 36 *Enterococcus* spp. (97%) were resistant to erythromycin, which is in accordance with the results of 86% enterococci either carrying *msrC* or *lsaA*, which is intrinsic to *E. faecalis* and confers low-level macrolide resistance [29,30]. No ARG associated with macrolides was observed in *E. casseliflavus* 64-1 and 53-1, indicating that a novel determinant could play a part in the erythromycin phenotype in 64-1 and 53-1. Notably, most infective *E. faecium* isolates are ampicillin resistance with an MIC of ampicillin of >16 μg/mL [31]. On the other hand, a strain that has an MIC of ampicillin of ≤2 μg/mL and lacks all IS*16*, *esp* and *hyl* genes should be regarded as safe [12]. Overall, 27 of 36 enterococci (75%) had an MIC of ampicillin of >2 μg/mL, and 8 *E. faecium* were found to have the mutations of *pbp5* conferring ampicillin resistance, which may be a potential risk factor associated with the prevalence and distribution of ARGs of probiotic enterococci.

The result that only a few isolates exhibited low-level resistance to vancomycin (8%) and ciprofloxacin (8%) is opposite to the majority of findings that have suggested that *E. faecalis* and *E. faecium* originated from the other sources, such as animals, water, and soil, have multidrug resistance phenotypes and high-level antimicrobial resistance. Among six known genes (*vanA*, *vanB*, *vanC*, *vanD*, *vanE*, and *vanG*) of glycopeptide resistance in *Enterococcus* spp., *vanA* is the most important and prevalent enterococcal ARG and confers high-level vancomycin and teicoplanin resistance to *E. faecium* and *E. faecalis* (namely VRE) [4]. No *Enterococcus* was positive for *vanA*, whereas *vanC*, conferring low-level vancomycin resistance [32], was detected in the less commonly encountered *E. casseliflavus* and *E. gallinarum* strains (Supplementary Table S3). Unexpectedly, 33 (2 *E. faecalis* and 31 *E. faecium*) of 36 enterococci were negative for *vanC*, which is an intrinsic trait of *E. casseliflavus* and *E. gallinarum* [33]. Generally, both *E. gallinarum* and *E. casseliflavus* are less pathogenic and show lower antimicrobial resistance than *E.* *faecium* and *E. faecalis*, but *E. gallinarum* and *E. casseliflavus* carrying *vanC* may influence host immunity, as suggested in recent reports [32]. No ARG conferring ciprofloxacin resistance was observed in 36 *Enterococcus* spp., suggesting that the ciprofloxacin resistance found in three enterococci may be associated with mutations in the quinolone resistance-determining region (QRDR) of the *gyrA* gene.

Intrinsic antimicrobial resistance of enterococci includes aminoglycosides [33], and no isolate with high-level gentamicin resistance (HLGR) or high-level streptomycin resistance (HLSR) was presented in this study. Generally, the frequency of HLGR of isolates that exhibited high rates of multiple antibiotic resistance was higher than HLSR in enterococci [34,35], and both HLGR and HLSR were much more frequently detected in VRE [36]. However, 30 of 31 *E. faecium* were positive to *aac(6′)-Ii*, which is intrinsic and specific for *E. faecium* and encodes an aminoglycoside 6′-N-acetyltransferase, conferring medium levels of most aminoglycosides [37].

**Figure 5:**
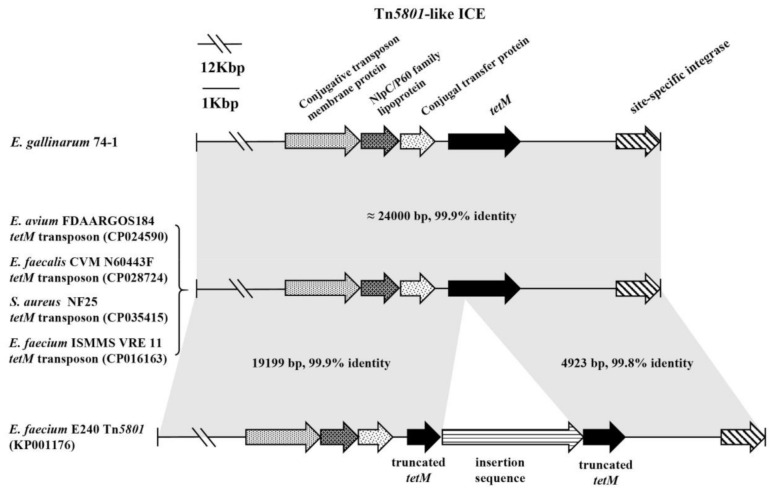
Tn *5801*-like ICE in *E. gallinarum* 74-1 compared with selected transposon ICEs (integrative conjugative elements) from VRE (vancomycin-resistant *Enterococcus)*, other enterococci, and *S. aureus.* The gray shading indicates regions sharing more than 99% DNA identity. The *tetM* gene was truncated into two parts in Tn*5801* of *E. faecium* E240.

Similarly, as reported in many studies [4], both the genotypes and phenotypes associated with erythromycin, vancomycin, and aminoglycoside resistance are intrinsic to enterococci. In contrast, only one *Enterococcus*, *E. gallinarum* 74-1, from an animal probiotic product, showed an intermediate level in the MIC (64 μg/mL) of tetracycline, and *tetM* was involved in acquired resistance of *Enterococcus* to tetracycline (Figure 4, Supplementary Table S3). The genetic environment of *tetM* in *E. gallinarum* 74-1 was further determined. The sequence of a chromosomal element of 24,265 bp containing the *tetM* gene and site-specific integrase was inserted between the gene encoding LPXTG cell wall anchor domain-containing protein (accession no. WP_142972242) and *guaA*, encoding glutamine-hydrolyzing GMP synthase, and the G + C content of this element was 35% (Figure 5), which is significantly lower than that (40.5%) of the entire genome of *E. gallinarum* ASM214049v1. This unique element, having a central region and carrying the gene *tetM*, the only ARG in the element, exhibited an overall nucleotide sequence identity of 99.9% to the *tetM* transposon of *E. avium* FDAARGOS184 (accession no. CP024590), *E. faecalis* CVM N60443F (accession no. CP028724), and *S. aureus* NF25 (accession no. CP035415) [38], and thus belonged to the Tn*5801*-like ICE (integrative conjugative element). Interestingly, Tn*5801* of *E. faecium* E240 (accession no. KP001176) integrated into *tetM*, resulting in *tetM* being truncated and showing no tetracycline phenotype (Figure 5). Tn*5801*, originally detected in *Staphylococcus aureus*, was a Tn*916* family element in which the genes *int* and *xis* in Tn*916* were replaced by a unique *int* gene (*int*_5801_) [39]. Importantly, a similar transposon ICE was also observed in VRE isolates (Figure 5) [38]. *Enterococcus*, particularly VRE, has the ability to transfer ARGs to produce frequent virulence characteristics, such as hemolysin, hyaluronidase, and adhesion [40].

In short, some enterococci of different origins may be from very diverse genetic lineages and environments, but the genus is capable of rapidly acquiring ARGs and mobile genetic elements, resulting in the dissemination of antibiotic resistance (AR) and ARGs among isolates, irrespective of their source or location.

### 3.5. Molecular Typing among Probiotic Isolates and Contaminated or Mislabeled Isolates

Among 33 *E. faecalis* and *E. faecium*, MLST yielded 13 sequence types (STs) (Figure 1), four of which, ST1615, ST1620, ST1616, and ST1694, were newly assigned in this study. No *E. casseliflavus* and *E. gallinarum* data were available according to the MLST database. All 13 STs, except for 15 singletons, were clustered into one clonal complex: CC94. Notably, all seven probiotic *E. faecalis* and *E. faecium* as active ingredients in human probiotic products shared the same STs (25 and 812, respectively), which were distinct from the STs of the other contaminated or mislabeled enterococci in probiotic products, indicating that these two particular sequence types belong to native probiotic isolates. MLST analysis of probiotic strains and isolates from probiotic products could thus be recommended to provide some indication as to whether the strain or isolate belongs to a problematic lineage.

## 4. Conclusions

This study provides comprehensive evidence for the hemolytic activity, label inaccuracy, high level of contamination of *E. faecium*, and the lack of active ingredients in probiotic products for human, animal, aquaculture, and plant use. Our findings also showed that virulence traits, a strong biofilm formation ability, and ARGs, particularly those carrying a transposon ICE, can be found in probiotic *E. faecalis* and *E. faecium* and other contaminated enterococci, irrespective of their origin, suggesting that such virulence factors and antimicrobial resistance determinants may transfer into the gastrointestinal system in humans and animals. Thus, in future worldwide applications of probiotics, our findings suggest that the probiotic potential of enterococci requires appropriate assessment to exclude the presence of virulence factors, antimicrobial resistance traits, and gene transfer risk, especially in countries where enterococci are commercialized as probiotic products.

## Figures and Tables

**Table 1:** The strength of biofilm formation among 36 *Enterococcus* spp.

Origin	Strong Biofilm	Moderate Biofilm	Weak Biofilm	Total
Human	11/17	0	0	11/17
Animal	9/11	0	1/11	10/11
Aquaculture	2/3	0	0	2/3
Plant	3/5	0	0	3/5
Total	25/36	0	1/36	26/36

## Data Availability

The draft whole genome sequence assemblies of the 36 *Enterococcus* spp. have been deposited in GenBank under BioProject PRJNA703331 https://www.ncbi.nlm.nih.gov/bioproject/PRJNA703331 (accessed on 3 March 2021).

## Supplementary Materials

The following are available online at https://www.mdpi.com/article/10.3390/microorganisms9040726/s1, Table S1: Information of commercial probiotic products, Table S2: Brief introduction of virulence factor function, Table S3: Genotype and phenotype of antimicrobial resistance in 36 *Enterococcus* spp. Isolates.
