# Supplementary material for: New Insights into the Virulence Traits and Antibiotic Resistance of Enterococci Isolated from Diverse Probiotic Products
Source: Microorganisms. 2021 Mar 31;9(4):726. doi: 10.3390/microorganisms9040726 (PMC8065695; doi:10.3390/microorganisms9040726)
Supplement: Supplementary file 1 [file microorganisms-09-00726-s001.pdf]

Supplementary data

Table S1. Information of commercial probiotic products.

Applicable targets	Origin	Product No.[a]	Purpose of application	Active ingredients[b]
Human	Fuyang, Anhui	1	Treatment of gastrointestinal disorder for adult	*Lactobacillus* spp., *Bifidobacterium* spp.
	Fuyang, Anhui	2	Treatment of gastrointestinal disorder for infant, adult and pregnant woman	*Lactobacillus* spp.
	Fuyang, Anhui	3	Treatment of gastrointestinal disorder for infant, adult and pregnant woman	*Lactobacillus* spp.
	Beijing	4	Treatment of indigestion and diarrhea for infant	***E. faecium***, *B. subtilis*
	Beijing	5	Treatment of indigestion and diarrhea for infant	***E. faecium***, *B. subtilis*
	Beijing	6	Treatment of gastrointestinal disorder for adult	***E. faecium***, *B. subtilis*
	Beijing	7	Treatment of indigestion and diarrhea for infant	***E. faecium***, *B. subtilis*
	Chaozhou, Guangdong	**8**	Treatment of gastrointestinal disorder for infant, adult and pregnant woman	*Lactobacillus* spp.
	Guangzhou, Guangdong	9	Treatment of gastrointestinal improvement for pregnant woman	*Lactobacillus* spp., *Bifidobacterium* spp., *Streptococcus* spp.
	Guangzhou, Guangdong	10	Treatment of gastrointestinal improvement for adult	*Lactobacillus* spp.
	Zhuhai, Guangdong	**11**	Treatment of gastrointestinal disorder for infant and pregnant woman	*Lactobacillus* spp., *Bifidobacterium* spp.
	Zhuhai, Guangdong	12	Treatment of gastrointestinal improvement for infant and adult	*Lactobacillus* spp., *Bifidobacterium* spp.
	Zhuhai, Guangdong	13	Treatment of gastrointestinal improvement for infant	*Lactobacillus* spp.
	Shijiazhuang, Hebei	14	Treatment of gastrointestinal improvement for infant and adult	*Lactobacillus* spp., *Bifidobacterium* spp.
	Shijiazhuang, Hebei	15	Treatment of gastrointestinal improvement for infant and adult	*Lactobacillus* spp., *Bifidobacterium* spp.
	Harbin, Heilongjiang	16	Treatment of gastrointestinal improvement for infant and adult	*Bifidobacterium* spp.
	Tsitsihar, Heilongjiang	17	Treatment of gastrointestinal improvement for infant and adult	*Lactobacillus* spp., *Bifidobacterium* spp., *Streptococcus* spp.
	Tsitsihar, Heilongjiang	18	Treatment of gastrointestinal improvement for infant and adult	*Lactobacillus* spp., *Bifidobacterium* spp., *Streptococcus* spp.
	Xinxiang, Henan	19	Treatment of gastrointestinal improvement for infant and adult	*Lactobacillus* spp., *Bifidobacterium* spp.
	Xinxiang, Henan	20	Treatment of gastrointestinal improvement for infant and adult	*Lactobacillus* spp., *Bifidobacterium* spp.
	Zhengzhou, Henan	21	Treatment of gastrointestinal improvement for infant and adult	*Lactobacillus* spp., *Bifidobacterium* spp., *Streptococcus* spp.
	Hohhot, Inner Mongolia	22	Treatment of gastrointestinal improvement for infant and adult	*Lactobacillus* spp., *Streptococcus* spp.

	Nanjing, Jiangsu	23	Treatment of allergy for infant and adult	*Lactobacillus* spp., *Bifidobacterium* spp., *Streptococcus* spp.
	Nanjing, Jiangsu	24	Treatment of gastrointestinal disorder for infant and adult	*Lactobacillus* spp., *Bifidobacterium* spp., *Streptococcus* spp.
	Nanjing, Jiangsu	25	Treatment of allergy for infant and adult	*Lactobacillus* spp., *Bifidobacterium* spp., *Streptococcus* spp.
	Suzhou, Jiangsu	26	Treatment of gastrointestinal improvement for infant and adult	*Lactobacillus* spp.
	Shenyang, Liaoning	27	Treatment of gastrointestinal improvement for infant	*Lactobacillus* spp., *Bifidobacterium* spp.
	Shenyang, Liaoning	28	Treatment of gastrointestinal disorder and diarrhea for adult	*B. licheniformis*
	Shenyang, Liaoning	29	Treatment of gastrointestinal disorder and diarrhea for infant	*B. licheniformis*
	Shenyang, Liaoning	30	Treatment of gastrointestinal improvement for infant	*Lactobacillus* spp., *Bifidobacterium* spp.
	Shenyang, Liaoning	**31**	Treatment of gastrointestinal disorder and diarrhea for infant	*B. licheniformis*
	Shenyang, Liaoning	32	Treatment of gastrointestinal disorder for adult	*B. licheniformis*
	Liaocheng, Shandong	33	Treatment of gastrointestinal improvement for adult	*Lactobacillus* spp.
	Qingdao, Shandong	34	Treatment of gastrointestinal improvement for infant and adult	*Lactobacillus* spp., *Bifidobacterium* spp.
	Qingdao, Shandong	35	Treatment of gastrointestinal disorder for infant	*Lactobacillus* spp.
	Zibo, Shandong	36	Treatment of gastrointestinal improvement for adult	*Lactobacillus* spp., *Bifidobacterium* spp., *Streptococcus* spp.
	Shanghai	37	Treatment of gastrointestinal improvement for infant and adult	*Lactobacillus* spp., *Bifidobacterium* spp.
	Shanghai	38	Treatment of indigestion and diarrhea for adult	*Lactobacillus* spp., ***E. faecalis***
	Jincheng, Shanxi	39	Treatment of constipation and diarrhea for adult	*Lactobacillus* spp., ***E. faecalis***
	New Taipei, Taiwan	40	Treatment of gastrointestinal improvement for adult	*Bifidobacterium* spp.
	Taipei, Taiwan	41	Treatment of gastrointestinal improvement for infant and adult	Lactic acid bacteria
	Yilan, Taiwan	42	Treatment of gastrointestinal improvement for infant	*Lactobacillus* spp.
	America	43	Treatment of gastrointestinal improvement for adult	*Lactobacillus* spp.
	Australia	44	Treatment of gastrointestinal improvement for adult	*Lactobacillus* spp.
	South Korea	45	Treatment of gastrointestinal improvement for pregnant woman	*Lactobacillus* spp., *Bifidobacterium* spp.
	South Korea	**46**	Treatment of gastrointestinal improvement for pregnant woman	*Lactobacillus* spp., *Bifidobacterium* spp.
	South Korea	47	Treatment of gastrointestinal improvement for infant	*Lactobacillus* spp.
Animal	Anhui, Hefei	48	Feed additive for livestock	-
	Beijing	49	Excreta degradation for livestock	-
	Beijing	**50**	Excreta degradation for poultry	-
	Beijing	51	Excreta degradation for poultry and livestock	-
	Chongqing	52	Feed additive for livestock	*B. subtilis*
	Daqing, Heilongjiang	**53**	Feed additive for livestock	*B. subtilis, B. licheniformis*
	Luoyang, Henan	54	Feed additive for swine and chicken	*B. subtilis, B. licheniformis,* lactic acid bacteria

	Luoyang, Henan	55	Feed additive for poultry and livestock	*Bacillus* spp., lactic acid bacteria, *Actinomyces* spp., Yeast
	Luoyang, Henan	56	Treatment of diarrhea for livestock	*Bacillus* spp., lactic acid bacteria, *Actinomyces* spp., Yeast
	Xinxiang, Henan	**57**	Feed additive for poultry	*Bacillus* spp., *Lactobacillus* spp.
	Zhengzhou, Henan	58	Feed additive for swine	*Bacillus* spp., lactic acid bacteria, Yeast
	Zhengzhou, Henan	59	Feed fermentation	*Bacillus* spp., lactic acid bacteria, Yeast
	Zhengzhou, Henan	**60**	Feed additive for poultry	*B. subtilis*, *Lactobacillus* spp.
	Zhengzhou, Henan	**61**	Feed additive for poultry and livestock	*B. subtilis*, ***Enterococcus* spp.**, lactic acid bacteria
	Zhengzhou, Henan	62	Feed additive for poultry and livestock	-
	Zhengzhou, Henan	63	Feed additive for chicken	***E. faecalis***
	Zhengzhou, Henan	**64**	Treatment of fungal infection for poultry and livestock	*B. subtilis*
	Changsha, Hunan	**65**	Feed additive for livestock	*B. subtilis,* ***E. faecalis***, *Lactobacillus* spp., Yeast
	Taizhou, Jiangsu	66	Feed additive for livestock	*B. subtilis, B. licheniformis, Lactobacillus* spp., *clostridium* spp.
	Taizhou, Jiangsu	67	Feed additive for swine	*B. subtilis, Lactobacillus* spp.
	Taizhou, Jiangsu	**68**	Feed additive for poultry and livestock	*B. subtilis*
	Taizhou, Jiangsu	69	Feed additive for livestock	*B. subtilis, B. licheniformis, Lactobacillus* spp.
	Xinghua, Jiangsu	**70**	Feed additive for poultry	*Bacillus* spp.
	Zhenjiang, Jiangsu	71	Treatment of diarrhea for poultry	*Bacillus* spp., lactic acid bacteria
	Nanchang, Jiangxi	72	Feed additive for poultry and livestock	*B. subtilis,* ***E. faecalis***, *Lactobacillus* spp., Yeast
	Xinyu, Jiangxi	73	Feed additive for poultry and livestock	*Bacillus* spp., *Bifidobacterium* spp., lactic acid bacteria
	Yichun, Jiangxi	**74**	Feed additive for poultry and livestock	*B. subtilis,* ***E. faecalis***, Yeast
	Yichun, Jiangxi	**75**	Feed additive for poultry	*B. subtilis, Pediococcus* sp., Yeast
	Taian, Shandong	**76**	Treatment of diarrhea for swine	*B. subtilis,* lactic acid bacteria
	Taian, Shandong	**77**	Feed additive for poultry and livestock	*B. subtilis,* lactic acid bacteria, Yeast
	Shanghai	**78**	Feed additive for rabbit	*Bacillus* spp., lactic acid bacteria, *Actinomyces* spp., Yeast, ***E. faecalis***
	Chengdu, Sichuan	79	Feed additive for poultry and livestock	*Bacillus* spp., ***Enterococcus* spp.**, *Lactobacillus* spp., Yeast
	Tianjin	**80**	Feed additive for poultry and livestock	*B. coagulans*
	Hangzhou, Zhejiang	81	Feed additive for pigeon	*B. subtilis, Lactobacillus* spp., Yeast
Aquaculture	Beijing	82	Water purification	-
	Zhengzhou, Henan	83	Water purification	*Bacillus* spp., lactic acid bacteria, Yeast
	Zhengzhou, Henan	**84**	Water purification	*B. subtilis*, *B. cereus*
	Zhengzhou, Henan	85	Water purification	-
	Zhengzhou, Henan	**86**	Water purification	*Bacillus* spp., lactic acid bacteria, Yeast

	Zhengzhou, Henan	87	Water purification	-
	Zhengzhou, Henan	**88**	Inhibit algae growth	*Bacillus* spp., lactic acid bacteria, *Actinomyces* spp.
	Zhengzhou, Henan	**89**	Water purification	*Bacillus* spp., lactic acid bacteria, Yeast
	Nanjing, Jiangsu	**90**	Water purification	*Bacillus* spp., *Lactobacillus* spp., *Nitrobacteria* spp., *Thiobacillus* spp.
	Nanjing, Jiangsu	**91**	Water purification	*B. subtilis*, lactic acid bacteria, *Nitrobacteria* spp.
	Xinyu, Jiangxi	92	Water purification	*Bacillus* spp., lactic acid bacteria, *Bifidobacterium* spp.
	Taipei, Taiwan	93	Water purification	*B. subtilis*
	Hangzhou, Zhejiang	**94**	Water purification	*Bacillus* spp.
	Zhoushan, Zhejiang	**95**	Water purification	*B. subtilis*
Plant	Beijing	**96**	Biocontrol	*Bacillus* spp.
	Beijing	**97**	Biocontrol and plant growth promotion	*Bacillus* spp.
	Shaoqing, Guangdong	98	Biocontrol	*P. fluorescens*
	Shaoqing, Guangdong	99	Biocontrol	*P. fluorescens*
	Harbin, Heilongjiang	100	Biocontrol	*P. fluorescens*
	Luoyang, Henan	**101**	Plant growth promotion	*B. subtilis, B. licheniformis*
	Nanyang, Henan	102	Biocontrol and plant growth promotion	*B. subtilis*
	Zhengzhou, Henan	103	Plant growth promotion	*Bacillus* spp., lactic acid bacteria, Yeast
	Zhengzhou, Henan	**104**	Biocontrol and plant growth promotion	-
	Yidu, Hubei	105	Plant growth promotion	*B. subtilis, B. licheniformis*
	Xinyu, Jiangxi	**106**	Biocontrol and plant growth promotion	*Bacillus* spp., lactic acid bacteria, *Bifidobacterium* spp.
	Binzhou, Shandong	107	Biocontrol	*P. fluorescens*
	Zhucheng, Shandong	108	Biocontrol	*P. fluorescens*
	Jinhua, Zhejiang	109	Biocontrol	*P. fluorescens*
	Netherland	110	Biocontrol and plant growth promotion	*B. subtilis*, *B. licheniformis*, *B. cereus, Actinomyces* spp.

Note: (a) Probiotic products exhibiting hemolysis were in bold and underlined. (b) -, the active ingredient was not identified in the product; Ingredients of product contained *Enterococcus* spp. were in bold and underlined.

Table S2. Brief introduction of virulence factor function.

Related factors	Function
Ace	Mediating binding to immobilized collagen type I, collagen type IV, and mouse laminin.
Acm	Interacts with collagen type I and to a lesser extent with collagen type IV.
Bee or Srt	Biofilm enhancer.
BopD	Homologous to a sugar-binding transcriptional regulator involved in biofilm production; The actual role is unknown, but the association of enhanced biofilm formation in the presence of glucose and the possible involvement of a sugar-binding transcriptional regulator suggest a linkage to increased biofilm production in *E. faecalis* in the presence of specific carbohydrates.
CdsA	Phosphatidate cytidylyltransferase catalyzes the synthesis of cytidine diphosphate-diacylglycerol, an essential phospholipid intermediate for the production of membrane phosphatidylglycerol and cardiolipin, contributes to capsule synthesis and daptomycin resistance.
Ebp	Ebp pili are important for adherence to host extracellular matrix proteins, including fibrinogen and collagen and play a role in biofilm formation.
EcbA	Binds to collagen type V.
EfaA	Might be functioning as an adhesion in endocarditis; A solute binding-protein receptor for manganese transport system.
Esp	Contributes to colonization and persistence of *E. faecalis* in urinary tract infections and also associates with promotion of primary attachment and biofilm formation of *E. faecalis* on abiotic surfaces.
Fsr	The Fsr quorum sensing system is an important regulator with both positive and negative effects, regulating *gelE*, *sprE* and *bopD* expression that are important for biofilm formation, along with genes implicated in several metabolic pathway.
Fss	microbial surface components recognizing adhesive matrix molecules.
GelE	Capable of degrading a broad spectrum of substrates, including casein, hemoglobin, collagen, fibrin, gelatin, certain *E. faecalis* sex-pheromone-related peptides and polymerized fibrin; May functioning as clearing the bacterial cell wall of misfolded proteins, and disruption of *gelE* gene has been shown to increase the bacterial chain length; May play a role in increasing dissemination of bacteria in high density environments; A *gelE* knockout has shown reduced virulence in models of mouse peritonitis, rabbit endophthalmitis, and in a Caenorhabditis elegans virulence model.
IS*16*	Transposase enriched in hospital-associated strains; contributes to the genomic plasticity of *E. faecium*.
Hyaluronidase (Hyl)	An important pathogenic bacterial spreading factor, and cleave hyaluronan, which is a constituent of the extracellular matrix of connective tissues; May also pave the way for deleterious effects of other bacterial toxins, thus increasing the magnitude of the damage; Another role may be to supply nutrients for the bacteria, since the degradation products of its target substrates are disaccharides that can be transported and metabolized intracellularly be bacteria.
orf2514 & orf2515	pseudogene encoding a surface B-type Cna protein, enhance adherence to extracellular matrix molecules.
PGC-1	PGC-1 is unique among Efm pilus loci as it also contains a housekeeping class A sortase and may play a role during colonization or pathogenesis in the mammalian host.
PGC-2	A pili cluster similar to Ebp from *E. faecium*.
PGC-3	may play a role during colonization or pathogenesis in the mammalian host but their biological functions remain to be determined.
PGC-4	putative role in biofilm formation and adhesion.

PtsD	PtsD encodes a sugar-specific membrane-associated EIID subunit required for carbohydrate transport; it is the first gene contributing to intestinal colonization in Efm during antibiotic treatment.
SagA	broad-spectrum binding to extracellular matrix (ECM) proteins, including fibrinogen, collagen type I, collagen type IV, fibronectin, and laminin.
Sal	Resistance to environmental stress and cell morphology.
Scm	Binds to collagen type V and fibrinogen.
SgrA	An LPxTG surface adhesin binds to fibrinogen and nidogen; May also play a role in adhesion to medical-device-related infections by forming a biofilm.
SprE	Contributes to pathogenesis in several infection models including Caenorhabditis elegans, mouse peritonitis, and a rabbit endophthalmitis model.
Swp	Novel class of cell surface proteins found in most Efm isolates. WxL proteins' involvement in binding human extracellular matrix proteins.
uppS	Contributes to host immune evasion and antibiotic resistance.

Table S3. Genotype and phenotype of antimicrobial resistance in 36 *Enterococcus* spp. isolates.

Isolate	ARGs	AR (μg/mL)[a]										
		Ampicillin	Erythromycin	Tetracycline	Tigecycline	Ciprofloxacin	Gentamicin	Streptomycin	Linezolid	Florfenicol	Vancomycin	Teicoplanin
38-1	*lsaA*	4	***1***	0.5	≤0.25	1	16	128	2	8	≤0.25	0.5
39-1	*lsaA*	2	***1***	0.5	≤0.25	1	8	64	2	8	≤0.25	0.5
1-1	*aac(6')-Ii* *msrC*	4	***8***	≤0.25	≤0.25	1	8	64	2	4	0.25	0.5
3-1	*aac(6')-Ii* *msrC*	4	***16***	≤0.25	≤0.25	0.25	16	64	2	8	0.5	0.5
4-1	*aac(6')-Ii* *msrC* *pbp5*	4	***16***	≤0.25	≤0.25	***4***	8	32	2	4	0.5	≤0.25
5-1	*aac(6')-Ii* *msrC* *pbp5*	4	***16***	≤0.25	≤0.25	1	8	32	2	4	0.5	0.5
6-1	*aac(6')-Ii* *msrC* *pbp5*	4	***16***	≤0.25	≤0.25	***2***	8	32	2	4	0.25	≤0.25
7-1	*aac(6')-Ii* *msrC* *pbp5*	4	***8***	≤0.25	≤0.25	1	8	32	2	4	0.5	0.5
12-1	*aac(6')-Ii* *msrC*	4	***16***	≤0.25	≤0.25	0.125	16	64	2	8	0.25	0.5
18-1	*aac(6')-Ii* *msrC*	4	***8***	≤0.25	≤0.25	0.25	64	64	2	4	0.25	0.5
25-1	*aac(6')-Ii* *msrC*	4	***8***	≤0.25	≤0.25	0.5	8	32	2	4	0.5	0.5
26-1	*aac(6')-Ii* *msrC*	4	***16***	≤0.25	≤0.25	1	8	64	2	8	1	≤0.25
28-1	*aac(6')-Ii* *msrC*	4	***8***	≤0.25	≤0.25	0.25	16	64	2	4	1	≤0.25
32-1	*aac(6')-Ii* *msrC*	4	***1***	≤0.25	≤0.25	0.5	8	128	2	8	<0.25	0.5
44-1	*aac(6')-Ii* *msrC* *pbp5*	4	***32***	≤0.25	≤0.25	1	4	32	2	4	0.5	0.5
45-1	*aac(6')-Ii* *msrC*	2	***16***	≤0.25	≤0.25	1	4	32	2	8	0.5	≤0.25
46-1	*aac(6')-Ii* *msrC*	4	***4***	≤0.25	≤0.25	1	8	32	2	8	0.25	0.5
54-1	*aac(6')-Ii* *msrC*	2	***16***	≤0.25	≤0.25	0.25	8	64	2	4	0.5	0.5
55-1	*msrC*	2	***32***	≤0.25	≤0.25	0.25	8	64	2	4	0.5	≤0.25
56-1	*aac(6')-Ii* *msrC*	2	***8***	≤0.25	≤0.25	0.5	4	32	2	8	0.25	≤0.25
65-1	*aac(6')-Ii* *msrC*	4	***8***	≤0.25	≤0.25	0.5	32	64	2	8	0.5	≤0.25
71-1	*aac(6')-Ii* *msrC* *pbp5*	2	***2***	≤0.25	≤0.25	1	16	64	2	4	0.25	≤0.25
73-1	*aac(6')-Ii* *msrC*	4	***16***	≤0.25	≤0.25	0.25	64	128	2	4	<0.25	0.5

76-1	*aac(6')-Ii*	*msrC*	*pbp5*	8	***16***	≤0.25	≤0.25	0.125	16	64	2	4	0.5	≤0.25
77-1	*aac(6')-Ii*	*msrC*		4	***16***	≤0.25	≤0.25	0.125	4	32	2	8	0.25	≤0.25
86-1	*aac(6')-Ii*	*msrC*		8	***16***	≤0.25	≤0.25	0.5	8	32	2	8	0.5	0.5
88-1	*aac(6')-Ii*	*msrC*		4	***16***	0.5	≤0.25	1	8	64	2	4	0.25	≤0.25
88-2	*aac(6')-Ii*	*msrC*		4	***8***	≤0.25	≤0.25	0.25	16	64	2	8	0.25	0.5
97-1	*aac(6')-Ii*	*msrC*		2	***8***	≤0.25	≤0.25	0.25	16	64	2	8	<0.25	≤0.25
102-1	*aac(6')-Ii*	*msrC*	*pbp5*	8	***1***	≤0.25	≤0.25	0.5	4	64	2	4	0.25	0.5
104-1	*aac(6')-Ii*	*msrC*		8	***16***	≤0.25	≤0.25	0.5	8	64	2	8	0.25	≤0.25
105-1	*aac(6')-Ii*	*msrC*		4	***16***	≤0.25	≤0.25	0.5	4	128	2	8	0.5	0.5
106-1	*aac(6')-Ii*	*msrC*		4	***16***	≤0.25	≤0.25	0.25	8	32	2	4	0.5	≤0.25
64-1	*vanC*			1	***1***	1	≤0.25	***2***	1	16	2	8	***8***	0.5
53-1	*vanC*			1	***4***	0.5	≤0.25	1	2	16	2	8	***8***	≤0.25
74-1	*vanC*	*tetM*		4	0.5	***64***	≤0.25	0.5	4	16	2	8	***8***	≤0.25

Note: (a) The MICs over the breakpoint were in bold and italic; no *Enterococcus* of high-level gentamicin resistance (HLGR) or high-level streptomycin resistance (HLSR) was found.
